# Autoimmune Hemolytic Anemia in Chronic Lymphocytic Leukemia: A Comprehensive Review

**DOI:** 10.3390/cancers13225804

**Published:** 2021-11-19

**Authors:** Francesco Autore, Raffaella Pasquale, Idanna Innocenti, Alberto Fresa, Federica Sora’, Luca Laurenti

**Affiliations:** 1 Dipartimento di Diagnostica per Immagini, Radioterapia Oncologica ed Ematologia, Fondazione Policlinico Universitario Agostino Gemelli IRCCS, 00168 Roma, Italy; idanna.innocenti@policlinicogemelli.it (I.I.); federica.sora@policlinicogemelli.it (F.S.); luca.laurenti@unicatt.it (L.L.); 2 Department of Oncology and Hemato-Oncology, University of Milan, 20122 Milan, Italy; raffaella.pasquale@unimi.it; 3 Sezione di Ematologia, Dipartimento di Scienze Radiologiche ed Ematologiche, Università Cattolica del Sacro Cuore, 00168 Roma, Italy; alberto.fresa01@icatt.it

**Keywords:** CLL, AIHA, steroids, rituximab, targeted drugs

## Abstract

**Simple Summary:**

This review analyzes the occurrence, clinical characteristics, and prognostic impact and treatment of autoimmune hemolytic anemia (AIHA) in chronic lymphocytic leukemia (CLL). Autoimmune hemolytic anemia is observed in about 10% of CLL. Pathogenesis is multifactorial involving humoral, cellular, and innate immunity, so the different mechanisms are well described in this review which also focuses on drugs associated to CLL-AIHA and on difficulties to diagnose it. There is a comprehensive revision of the main published casistics and then of the treatments; in particular the paper analyzes the main chemo-immunotherapeutic agents used in this setting. Since the therapy depends on the presence and severity of clinical symptoms, disease status, and comorbidities, treatment is nowadays more individualized in CLL and also in CLL-AIHA. Patients not responding to corticosteroids and rituximab are treated with CLL-specific drugs as per current guidelines according to age and comorbidities and new targeted agents against BCR and BCL-2 which can be given orally and have few side effects, are very effective both in progressive CLL and in situations such as AIHA.

**Abstract:**

Chronic lymphocytic leukemia (CLL) patients have a greater predisposition to develop autoimmune complications. The most common of them is autoimmune hemolytic anemia (AIHA) with a frequency of 7–10% of cases. Pathogenesis is multifactorial involving humoral, cellular, and innate immunity. CLL B-cells have damaged apoptosis, produce less immunoglobulins, and could be responsible for antigen presentation and releasing inflammatory cytokines. CLL B-cells can act similar to antigen-presenting cells activating self-reactive T helper cells and may induce T-cell subsets imbalance, favoring autoreactive B-cells which produce anti-red blood cells autoantibodies. Treatment is individualized and it depends on the presence and severity of clinical symptoms, disease status, and comorbidities. Corticosteroids are the standardized first-line treatment; second-line treatment comprises rituximab. Patients not responding to corticosteroids and rituximab should be treated with CLL-specific drugs as per current guidelines according to age and comorbidities. New targeted drugs (BTK inhibitors and anti BCL2) are recently used after or together with steroids to manage AIHA. In the case of cold agglutinin disease, rituximab is preferred, because steroids are ineffective. Management must combine supportive therapies, including vitamins; antibiotics and heparin prophylaxis are indicated in order to minimize infectious and thrombotic risk.

## 1. Introduction

### 1.1. CLL

CLL is one of the most common types of leukemia in the western world, representing approximately 20% of all hematological diagnoses [1]. The median age at diagnosis is between 67 and 72 years. CLL is a malignant lymphoid neoplasm characterized by progressive accumulation of functionally incompetent lymphocytes, which are usually monoclonal in origin. CLL has an extremely heterogeneous clinical course, ranging from years of stable disease to rapidly progressive disease [2]. Historically, CLL patients are defined using Rai and Binet, so their risk and prognosis have been calculated. Early-stage asymptomatic CLL patients do not require immediate therapeutic intervention but only observation; treatment is necessary for patients with advanced disease or when “active disease” is observed. “Active disease” criteria are progressive bone marrow failure manifested by anemia and/or thrombocytopenia; bulky, or progressive, or symptomatic hepato-splenomegaly and/or lymphadenopathies; progressive lymphocytosis with an increase of more than 50% in 2 months or rapid lymphocyte doubling time (LDT); symptomatic or functional extranodal involvement (skin, kidney, lung, spine); autoimmune complications not controlled by steroids; constitutional symptoms such as significant fatigue, night sweats for more than 1 month, unintentional weight loss more than 10% during the previous 6 months, fevers for more than 2 weeks without evidence of infection [3,4,5].

### 1.2. Autoimmunity

Autoimmunity has a genetic background represented by HLA genotype, cytokine polymorphisms, and environmental predisposition that is defined by infectious agents, neoplastic clones, and medical or cellular treatment; it derives from an imbalance between pathogenic factors generated by autoreactive T and B cells and regulatory factors that control the immune response. In particular, it results from the loss of both peripheral and central tolerance against self-antigens. The first is the mechanism by mature T cells recognizing self-antigens in peripheral tissues become unable to respond to a subsequent encounter with these same antigens. It is responsible for maintaining the tolerance of T lymphocytes towards tissue-specific autologous antigens. The second selects immature lymphocytes in the primary lymphoid organ. It derives from a selection in the thymus and bone marrow, where lymphocytes can meet only a part of self-antigens. For T cells, the thymus is the locale for the establishment of central tolerance and the bone marrow is the locale for B cells. Central tolerance may not be complete and autoreactive cells, in particular B cells, can emerge in the periphery following somatic mutation. After interaction with self-antigens, B-cells endure clonal deletion by way of apoptosis and/or anergy induction and T-cells may become tolerogenic. They may become regulatory T cells (T-regs) and suppress self-reactive T cells, down-regulate interleukin-2, and secrete other cytokines [6,7,8].

### 1.3. CLL and Dysregulation

CLL patients have defects in humoral and cell-mediated immunity, represented by hypogammaglobulinemia, and functional alteration in T cell subsets, complement, neutrophils, and monocytes. CLL B-cells allow activation and propagation of immune dysregulation through immunosuppressive cytokines or by downregulation of surface molecules. Thus, defects in immunoglobulins class switching and B-cell function generate a progressive hypogammaglobulinemia that is typical for CLL patients, in particular regarding subtypes IgG3 and IgG4 generating risk of recurrent infection sustained by encapsulated organisms. Moreover, in a CLL progression setting, there is an expansion of circulating T cells that may be anergic, and with compromised proliferative potential, but able to produce cytokines [7,8]. Immune dysregulation generates a raised risk of cancers and opportunistic infections such as herpes zoster virus and cytomegalovirus and also autoimmune phenomena, in particular cytopenia, in CLL patients [8].

## 2. CLL and AIHA

Growing evidence since 1960s highlights how blood components are the main target of autoimmunity in CLL. The most frequent autoimmune cytopenias (AICs) are autoimmune hemolytic anemia (AIHA, 7–10%) and immune thrombocytopenia (ITP, 1–5%), while pure red cell aplasia (<1%) and autoimmune neutropenia (0.17%) are unusual. AIHA can present in up to a third of patients with CLL during the course of their disease, while in 10–15% cases at diagnosis [9,10,11,12].

### 2.1. Pathogenesis of AIHA

From a clinical point of view, AIHA is a heterogeneous condition, from fully compensated to life threatening. It is caused by autoantibodies directed against red blood cells (RBCs), with or without complement activation. In general, AIHA can be primary (idiopathic, 50%) or secondary to an underlying condition, such as lymphoproliferative disease (20%), infections (20%), immunodeficiency, and cancer.

The mechanisms for RBC destruction may be represented by intravascular or extravascular hemolysis. AIHA is defined according to autoantibody thermal characteristics [13,14,15]. Warm AIHAs (wAIHAs) is 70–80% of cases; involved antibodies are polyclonal IgG and direct antiglobulin test (DAT) is positive for IgG or IgG plus complement fraction 3d (C3d). This type of AIHA is called warm because antibodies act at 37 °C. Density of these RBC antigens is usually not high enough to fix complement. Macrophages clear opsonized RBC in extravascular sites [15]. Cold AIHAs (cAIHA) are usually sustained by monoclonal IgM able to fix complement at low temperatures generating complement-mediated RBC intravascular lysis and DAT is usually positive only for C3d. A small number of patients have a mixed form of AIHA with a positive DAT for both IgG and C3d indicating presence of warm IgG autoantibodies and high titer cold agglutinine. Cold agglutinin disease (CAD) is due to clonal or oligoclonal IgM antibody binding to RBC antigens at low temperatures. Complement binding is defined by IgM structure and high antigen density on RBC, with RBC aggregation and complement activation. This may be complete causing intravascular hemolysis or incomplete with extravascular one [16,17]. AIHA pathogenesis involves humoral, cellular, and innate immunity. As regard the first, polyclonal high-affinity IgG autoantibodies produced by non-malignant B cells are fundamental. Auto-antibodies, usually IgM type, can also be produced by CLL B cells. They may interfere not only with mature B cells but also with precursor maturation. For the second type, T cells are considered essential. CLL-B cells and T cells in the context of CLL express altered patterns of surface molecules and CLL patients present an imbalanced cytokine environment. CLL B-cells have impaired apoptosis and immunoglobulins production, and they may present antigen and release inflammatory cytokines. A T-cell subsets imbalance may cause the emergence of autoreactive B-cells producing anti-RBC. T-reg expansion could reduce anti-tumor immune response and compromise immunosurveillance. A decrease in TLR2 and TLR4 and an increase in TLR9 expression are described in CLL patients. Regarding biological CLL parameters, unmutated immunoglobulin heavy-chain variable region gene (IGHV) status, stereotyped IGHV frames, and unfavorable cytogenetics represented by chromosome 17p and/or 11q deletions and nine down-regulated miRNAs are risk factors for developing AIHA in the context of CLL [7,18].

### 2.2. Drugs Associated with CLL-AIHA

Moreover, several drugs are associated with AIHA through different mechanisms. There are drug-independent antibodies and drug-dependent antibodies that are able to activate an immune response only in the presence of the drug. Several drugs such as cladribine, methyldopa, and fludarabine may generate AIHA by way of adsorption, immune dysregulation, and other unknown systems. Robak T et al. described a group of 114 CLL patients treated with cladribina. DAT-positive AIHA was detected in 25 (21.9%) patients, in 23 cases before and 2 patients after starting treatment with the drug. Six patients obtained complete remission of hemolysis and 8 patients obtained partial remission; 11 patients did not respond. Two patients without a history of hemolysis developed AIHA during treatment: one patient died because of CNS hemorrhage and the other obtained remission with steroids and chlorambucil. According to these authors, cladribine could both suppress and trigger AIHA in CLL patients [19]. Fludarabine, a historic drug in CLL treatment, induces an imbalance between Th17 and T-regs as an additional AIHA pathogenic mechanism. If fludarabine is associated with cyclophosphamide and rituximab, patients don’t develop AIHA, thanks to immune-suppression and autoreactive T-cell reduction by these drugs [20]. In UK LRF CLL4 trial, that is the largest prospective trial in CLL to study prognostic role of both a positive DAT and AIHA, 777 patients were randomized to receive chlorambucil or fludarabine, as a single agent or in combination with cyclophosphamide (FC). Patients treated with chlorambucil or fludarabine developed AIHA more than twice as those receiving FC. Four patients died for AIHA during single-agent fludarabine treatment. DAT-positive CLL patients may benefit from FC or FC plus rituximab rather than fludarabine or chlorambucil monotherapy [21]. Purine analogs, such as bendamustine, cause a drug-independent AIHA: patients acquire a circulating drug-induced antibody at the first contact with the drug and may undergo acute reactions at following administrations, with IgG and/or C3 DAT positivity. Risk factors for bendamustine-induced AIHA include diagnosis of CLL and previous purine analog treatment [12,22,23,24,25,26,27,28]. New small molecules such as Bruton’s tyrosine kinase (BTK) inhibitor ibrutinib, the BCL-2 inhibitor venetoclax, and the Phosphoinositol-3-kinase (PI3K) inhibitor idelalisib, are rarely associated with AIHA. Ibrutinib and venetoclax are able to inhibit autoantibodies producing B cells and act on T-cell homeostasis, so there are some reports regarding the remission of AIHA during treatment with these molecules. Idelalisib is associated with autoimmune hepatitis, colitis, and pneumonitis, but rarely with AIC [29,30].

### 2.3. Diagnosis of CLL-AIHA

In the context of CLL, the diagnosis of AIHA could be difficult because blood parameters such as hemoglobin, hemolytic markers, and DAT which are relevant for AIHA may be altered by CLL progression or concomitant treatment. Regarding anemia, it could be secondary to bone marrow infiltration or failure, gastrointestinal blood loss secondary to use of corticosteroids, thrombocytopenia, mucositis or coagulopathy, hypersplenism, vitamin or iron deficiencies, renal disease, and marrow suppression secondary to chemotherapy. Regarding hemolytic signs, LDH may be elevated for CLL progression, haptoglobin for inflammatory response, bilirubin levels for treatment, and reticulocytosis could be absent or inadequate for bone marrow infiltration or suppression by cytokine and/or anti-erythroblasts antibodies and chemotherapy. Regarding the DAT, it is not enough to diagnose AIHA because it may be positive in normal subjects, also without hemolysis. Thus, around 10% of patients can present negativity of DAT but they show clear evidence of AIHA, probably due to the low-affinity or to very small autoantibodies titer [7].

### 2.4. Main Experiences of CLL-AIHA

Many authors have described the association of AIHA and CLL; major findings are summarized in Table 1.

An Italian monocentric retrospective study by Mauro FR et al. [31] in 2000 analyzed a cohort of 1203 CLL patients, of whom 52 patients (4.3%) developed AIHA, 45 wAIHA, and 7 cAIHA. Lymphocyte count > 60 × 10^9^/L, age > 65 years, and male gender were independent parameters correlating with increased rate of AIHA at CLL diagnosis. Patients previously treated with chlorambucil plus prednisone and with fludarabine plus prednisone showed a similar rate of AIHA (1.8% and 2.5%, respectively). IgG AIHA and concomitant diagnosis of CLL and AIHA were identified as independent factors significantly correlated with a better survival in AIHA-CLL patients. Barcellini et al. [32] in 2006 conducted a multicentric prospective and retrospective study to analyze the relationship between autoimmune manifestations and CLL stage and therapy; they collected 3150 Italian CLL patients and the most frequent form of AIC was AIHA (129 cases, 66%). CLL-associated AIHA in 89% of cases were sustained by warm autoantibodies and only 11% of cases were cold hemagglutinin diseases due to IgM autoantibodies. Age over the median, stage C, and 1st or 2nd-line therapy were identified as independent risk factors by multivariate analysis. Duek et al. [33] in the same period analyzed 964 patients of the Israel CLL Study Group, and they found 115 CLL patients with autoimmune disorders. Also in this group, the main autoimmune disorder was AIHA, reported in 55 patients (5.7%). Their data showed that activated lymphocyte morphology, high levels of IgG and beta-2-microglobulin, and CD38 and/or FMC7 increased expression may raise the risk of an autoimmune disorder in a CLL setting. In a study by Zent et al, [34] 74 (4.5%) CLL patients developed autoimmune disease, AIHA was observed in 55% of patients, while AIHA and ITP (Evans syndrome) was observed in 10%. Forty-one patients (55%) developed AIC prior to CLL treatment. Only 9 out of the 33 patients who experienced autoimmune complication after CLL treatment had received purine analogs. Male gender, unmutated IGHV, specific BCR subsets, and treatment necessity have been associated with AIC risk. According to Moreno et al. [35] AIC are correlated with high peripheral lymphocyte count, short LDT, high serum beta-2-microglobulin levels, and high ZAP-70 and CD38 expression, but not with treatment type represented by fludarabine and alkylating. From a prognostic point of view, AIC did not significantly influence prognosis. Patients with advanced disease related to an immune complication had a better prognosis than patients in whom advanced stage reflected a high tumor burden only. Different outcome of patients with advanced disease according to cytopenia type, as shown in their and other studies, makes a case for including a stage C immune group in CLL prognostic stratification. Shvidelet al. [36] analyzed a large cohort of 1477 CLL patients, and 80 patients had AIHA Patients with AIHA from the time of CLL diagnosis had significantly shorter survival than those without anemia and OS was similar for patients with AIHA or anemia secondary to bone marrow infiltration. AIHA has a significant negative impact on CLL patients survival. Almost all the patients with AIHA had CLL progression. Thirty-three patients were treated before developing AIHA with alkylating agents, fludarabine-containing regimens, or both. Four patients developed AIHA during the first 3 cycles of fludarabine monotherapy or fludarabine plus cyclophosphamide. Demir et al. [37] analyzed 192 CLL patients and 8 patients experienced AIHA. A much older age and CLL advanced stage were observed in patients with AIC. Visco et al. [39] in 2014 reported data of AIC in CLL: AIHA is more frequently associated with patients with unfavorable CLL biological risk factors such as del11q, del17p, and unmutated IGHV. AIHA secondary to CLL respond less favorably to standard treatments than their primary forms and treatment of underlying CLL could often be necessary. Laurenti et al. in 2015 [25] collected 235 patients affected by CLL treated with bendamustine-rituximab (BR). AIHA was detected in 4.35% of patients receiving BR as a second-line treatment and in none receiving BR as first-line treatment. A greater AIHA risk was observed in patients with aggressive disease and poor prognostic features, such as unfavorable cytogenetics and unmutated IGHV. Increased risk of AIHA was detected in patients with history of autoimmune phenomena or front-line therapy with fludarabine, probably for its structure similar to bendamustine. AIHA is probably due to CD4 cells depletion which can lead to failure to control auto-reactive T-cells that are free to create autoimmunity. The greater incidence of AIHA in patients receiving BR as second-line compared to those receiving BR as first-line is most likely due to greater immune disturbances associated with more aggressive disease and repeated rounds of treatment. This scheme could however be safely administered even to patients with a previous history of AIHA or a positive DAT. Visentin et al. [8] in 2017 investigated the most common and significant complications in patients affected by CLL: major infections, secondary cancers, and autoimmune diseases. These complications were described as mutually exclusive. A patient with one complication has a very low risk to develop the other two; only 7 (0.9%) subjects experienced all three complications. Outcome of patients with autoimmune diseases is similar to patients without any complication, while the prognosis of patients with major infections is dismal. Female gender, advanced stage disease, previous treatment, 11qdel, CD38 positive, and CAD are associated with an increased risk of autoimmune diseases. Atef et al. [38] analyzed 101 CLL Egyptian patients: prevalence of AIC was 11.9% and AIHA was the most common type. Patients with AIC showed a lesser response to treatment. No significant difference in overall survival was found between patients without AIC and those with AIC or with immune and combined or infiltrative cytopenia. Patients with AIC showed significantly less CLL bone marrow BM infiltration compared with patients without AIC. A significantly higher percentage of patients without AIC (64.3%) had a diffuse infiltration pattern of the BM compared with 38.7% of patients with AIC.

## 3. Treatment

Treatment in CLL-associated AIHA is individualized and it depends on the presence of clinical symptoms (acuteness of the onset, grade of anemia, and degree of hemolysis) and their severity, disease status, and concomitant comorbidities. Anemia, if symptomatic, is primarily an indication for therapy in newly diagnosed and also persistent AIHA. [7,13,24]. Lower tolerance for anemia is typical of elderly patients who therefore more frequently require treatment, and whose adverse drug reactions, drug interactions, and therapy toxicities are more common. RBC transfusions are usually indicated in critical cases with deeper levels of hemoglobin (usually Hb < 6 g/dl) and/or symptomatic anemia, if hemodynamically unstable. To reduce the risk of alloimmunization blood transfusion should be administered only if indicated. In CLL cases, the requirement could be higher than primary cases due to BM impairment and inadequate reticulocytosis. It is also necessary to exclude the presence of alloantibodies before giving red cells. In critical cases, it is not possible to avoid or delay transfusion because of uncertainty in matching, but immediate corticosteroids treatment should be administered. In cases of CAD, warming coils to transfuse blood should be used [14,16,17,40,41]. In emergency situations, although the role of blood transfusion is crucial, immunoglobulins (0.4 g/kg for 5 days or 1 g/kg for 2 days) could be used as a bridging treatment but immunosuppressive drugs could be added. Similar to other autoimmune diseases, the administration of a bolus of intravenous methylprednisolone 500 mg may be used in fulminant and severe situations. Methylprednisolone boli is an option with or without intravenous immunoglobulins, in patients with signs of acute hemolysis and slow response to steroid treatment [39,42]. By analogy with ITP in which is possible to use thrombopoietin receptor agonists, the transient and off-label use of an erythropoiesis-stimulating agent (ESA) at a high dose may be added in patients with severe AIHA, with a high need of blood transfusions and reticulcytopenia [24,43,44,45]. Finally, management of AIHA if CLL-associated must consider the stage of the hematological malignancy: in patients with stage A CLL it is the same as AIHA, whereas in patients with active CLL, it is the treatment of the neoplastic disease.

Here, we present different treatment options according to literature data on wAIHA and cAIHA. Principal options are shown in Table 2 and Table 3.

### 3.1. Warm AIHA

#### 3.1.1. Warm AIHA First Line Treatment

Steroid treatment is usually the first line: the most chosen is prednisone initially at 1 mg/kg/day for 3–4 weeks and then slowly tapered. It is necessary to taper after 14–21 days in responsive patients but also in unresponsive patients by 21 days. For patients responding to the treatment, you should consider stopping it after at least 3 months after a complete response was achieved. Corticosteroids must be tapered because side effects, in particular corticosteroid-induced diabetes, worsening of pre-existing diabetes, gastrointestinal bleeding, osteoporosis, fractures, and osteonecrosis of the femoral head, are cumulative: most patients became symptomatic if a dose of 1 mg/kg daily is continued for more than 4 weeks. It is important to stop steroids because of long-term toxicities but in case of refractoriness it may be appropriate in general practice to administer long-term therapy with low doses of prednisolone (usually ≤ 10 mg daily) to effectively control AIHA, but long-term remissions are less [46,47,67]. When there is no improvement after about the first three weeks of treatment, other medications should be added. Alternative glucocorticoids such as dexamethasone could have therapeutic role, but there is no evidence of comparable outcomes [41,48]. When relapsing, it is usually administered as a steroid rescue, for its rapid effect, but at the same time a second-line treatment should be started.

The combined therapy of rituximab to steroids, also in first-line treatment, shows a better response rather than steroids monotherapy [47,49,50]. The addition of rituximab reduces the number of repeated cycles of steroids, and this is particularly useful in old patients with comorbidities. Therefore, predniso(lo)ne plus rituximab may be an option for selected patients with severe AIHA or in elderly patients with comorbidities.

#### 3.1.2. Warm AIHA Second Line Treatment

A second-line treatment should be considered when the patient is unresponsive or he relapses as the steroid is weaned. In cases of an early stage of CLL, management of AIHA is the same as in primary AIHA, in cases of active CLL you should consider CLL therapy as per current guidelines according to age and comorbidities [24,68]. This aggressive approach could be avoided in steroid-refractory wAIHA without signs of CLL progression. In this exception, it is possible to administer rituximab monotherapy [50]. Parameters such as patient age/comorbidities and CLL molecular characteristics and potentially hemolytic side effects should guide the choice between chemoimmunotherapy and small molecules [4]. Fludarabine or chlorambucil as single agents are not the best options in this setting, in particular, fludarabine as an inductor of AIHA or pure red cell aplasia even if this effect seems to be reduced if rituximab is administered together. CLL-associated wAIHA could be managed by combination regimens such as rituximab, cyclophosphamide, and dexamethasone (RCD), rituximab, cyclophosphamide, vincristine, and prednisolone (R-CVP) [40,59,60,61] or BR [28,54].

Previous studies showed high (>80%) and long-term response rates: in particular 89% and 100% were obtained with RCD [60,69], with a median response time of 24 months [59]; 95% with R-CVP with 70% of complete responses and a median response time of 21.7 months [61] and 80% with BR with a time to next treatment of 28.3 months [28].

New targeted drugs (BTK inhibitors and anti BCL2) are usually used in the second-line treatment of CLL, showing excellent efficacy but could be used in first-line in or outside clinical trials; in this way, they are a real option for the management of CLL-associated AIHA, alternative to standard chemo-immunotherapy. They must be preferred in patients with TP53 aberration and should be considered in refractory cases. Even if there are no specific studies on their role in the setting of CLL with AIHA, it is real practice to use them after or together with steroids to manage AIHA because they exert a beneficial impact on CLL-related pre-existing AIHA with an effective control of the autoimmune phenomena. Moreover, AIHA seems to lose its prognostic significance widely demonstrated in the era of chemoimmunotherapy; AIHA seems to lose the impact on the outcome of patients treated with targeted drugs [30]. In particular, ibrutinib was demonstrated to be safe in patients showing AIHA in CLL with progressive disease [70,71]. Ibrutinib determined an improvement in a good rate of patients with pre-existing AIHA and induced a low rate of treatment-emergent AIHA. Moreover, cases of successful treatment of AIHA with ibrutinib have been reported both in high-risk patients and in standard-risk patients with refractory AIHA [70,72,73,74]; in particular the revision of 301 cases from four different clinical trials [71] demonstrated that all patients controlled AIHA when ibrutinib was administered and 86% could discontinue specific treatment for AIHA after a median time of 4.7 months. Also acalabrutinib, a new BTK inhibitor recently approved for relapsed/refractory CLL, should reduce concomitant AIC including AIHA [75]. PI3K inhibitors, which usually triggered autoimmune phenomena, seemed to register partial response on secondary wAIHA [76]. Data on idelalisib reported 95% of AIC response rate and 66% of discontinuation treatment for AIC [56]. As far as venetoclax concerned, some experiences of success have been published. For example, a CLL patient who showed del17p and refractory AIHA obtained a response with venetoclax after three months of treatment. Its role in secondary wAIHA has been displayed, but not all the experiences were successful [57,58].

#### 3.1.3. Warm AIHA Treatment for Refractory Cases

Other possible options for refractory cases include alemtuzumab, cytotoxic immunosuppressor, other monoclonal antibodies, and splenectomy.

Alemtuzumab has not been used yet for its infectious and autoimmune complications [51,62].

Cytotoxic immunesuppressors, such as cyclophosphamide, azathioprine, cyclosporine, and mycophenolate, showed heterogeneous but in general weak efficacy also in primary AIHA and so rarely they are used in CLL secondary AIHA [14,63].

New generation monoclonal antibodies, such as ofatumumab and obinutuzumab, may have efficacy secondary AIHA [52]. Splenectomy is an invasive and irreversible procedure with an increased risk of thrombosis and encapsulated bacterial infections, in particular in patients heavily pre-treated for CLL [77]. In case of patients failing second-line treatment, it is necessary to refer them to tertiary experienced centers, especially for novel agents and clinical trials, whenever possible.

### 3.2. Cold AIHA

Patients with cAIHA may have a less serious clinical presentation because of higher Hb levels (usually > 9 g/dL) and symptoms associated with cold agglutinin (acrocyanosis, itch, and urticarial the most common); therefore, they could sometimes require only a watch and wait approach. Treatment is preferred when there is transfusion-dependency, active hemolysis (even if LDH cannot be the unique parameter to evaluate because it is usually increased in CLL), and invalidating cAIHA symptoms. Patients who suffer from mild to moderate anemia often showed exacerbation at cold temperatures, and even if they do not require therapy these levels are usually associated with reduced quality of life. These patients can try to manage them with thermal protection only, but if it fails they may require treatment [54]. Sometimes, exacerbations can occur during bacterial infections or other febrile episodes and in these cases short-term transfusion support without a long-term systemic treatment can work [17,78]. If bacterial infections are treated early, it may prevent hemolytic crisis. In rare cases, patients with CAD could require repeated transfusions although they are treated with a high dose of corticosteroids and rituximab, and they should be hospitalized in the intensive care unit. Immunoglobulins and/or plasma exchange can be considered to reduce transfusion support [78]. In particular, plasma exchange can reduce the level of the IgM monoclonal protein, which is found intravascularly; the procedure may potentially provide transient benefit in urgency or if other options are not available [79]. Even if without prospective studies, exchange of the plasma volume with albumin daily or every other day is recommended [80].

Treatments used in wAIHA are not effective and are not recommended in CAD [81]. Corticosteroids can be effective only if administered at high doses. When analyzing refractory patients, current guidelines suggest the introduction of a CLL-directed therapy. Patients with severe CAD who need treatment if they are fit may be treated with chemo-immunotherapy such as BR regimen; if they are unfit, the recommended first-line treatment is rituximab single agent.

Sometimes hemolytic anemia can become severe and life-threatening due to acute episodes of infections, major surgery, or severe cold exposure [78]. Usually responses to treatment with rituximab in combination with bendamustine or not are slow [60], so blood transfusions could be given in this period. Eculizumab, blocking the terminal complement pathway and intravascular hemolysis [65,66], could be an option even if it may take a long time to work [82,83]. Rituximab monotherapy 375 mg/m^2^ weekly for 4 weeks is a treatment option for CAD with good overall response rates [39,50,53]. The addition of rituximab should be considered, together with a quick steroid reduction as soon as Hb has been stabilized. There have been described few toxicities with rituximab and this course can be repeated and it is often effective in relapsed CAD [4,54]. In a recent trial regarding BR, 45 patients received rituximab 375 mg/m^2^ day 1 and bendamustine 90 mg/m^2^ day 1 and 2 for 4 monthly cycles [54]. Seventy-one per cent had good responses (40% complete response and 31% partial response) with 20% of grade 4 neutropenia and only 11% of infection with or without neutropenia. Splenectomy is not recommended in CAD because it is ineffective, as the sensitized RBC are mainly removed in the liver, the main location in extravascular hemolysis. In the second-line situation, Br is recommended if not administered in first-line or contraindicated [54]; it can also be repeated if the relapse happened at least two years from the first course. Rituximab monotherapy can be repeated if the response lasted at least one year [84]. In addition, rituximab plus fludarabine (rituximab 375 mg/m^2^ on days 1, 29, 57, and 85, and fludarabine orally, 40 mg/m^2^ on days 1–5, 29–34, 57–61, and 85–89) showed good responses [55]; fludarabine-induced wAIHA did not occur, but transient, mild exacerbations of CAD worsened by infections [55,78] and late-occurring hematologic malignancies were reported [85]. Good results have also been reported with the use of oral fludarabine, but mainly in primary cAIHA cases [55].

There is no standardized third-line treatment; inclusion of patients in clinical trials, if available, is necessary. Many studies are based on the use of BTK inhibitors ibrutinib and acalabrutinib, which act for reducing the IgM monoclonal protein [85]. BTK inhibitors seem to be effective in refractory CAD: if clonal cells that produce CA usually do not express the typical MYD88 L265P mutation, it means that this mutation is probably not essential for their effect [86]. Ibrutinib was recently administered in a few patients with cAIHA secondary to CLL [87], moreover, it is approved for the treatment of Waldenström macroglobulinemia in which CAD is frequent; acalabrutinib showed activity in CLL patients [64]. Moreover, venetoclax, a BCL2 inhibitor, has been described with a role in this treatment [88,89]. Other clinical trials concerned about complement inhibitors that eliminated the fixation of complement on the red cell surface. Eculizumab, the monoclonal anti-C5 antibody, was the first used from this class in the cases of severe CAD [65,66], but in theory more upstream pathway inhibition should work better [90,91,92]: sutimlimab (BIVV009, TNT009), ANX005, an inhibitory molecule against C1q [93] and pegcetacoplan (APL-2), are studied for this intent.

### 3.3. Supportive Management

Thromboprophylaxis has the indication in AIHA patients to minimize their risk of venous thromboembolism (VTE), greater both in wAIHA and cAIHA from published experiences [46,94,95,96]. VTE prophylaxis should be prescribed in all hospitalized patients without a contraindication, especially in terms of platelets and coagulation parameters [7,22], and in ambulatory patients in case of active hemolysis or if additional risk factors for VTE are present [97]. Active hemolysis, which is represented by more severe anemia, high reticulocyte count, and high LDH levels, has been associated with an increased rate of VTE. Each patient should be studied for disease-specific risk factors, above mentioned, and general risk factors for VTE (age above 70, active neoplasia, previous VTE, immobilization, thrombophilia, recent trauma and/or surgery in particular splenectomy, cardiac and/or respiratory failure, infection) to determine the exact risk. Positivity for anti-cardiolipin antibodies or lupus anticoagulant is a controversial risk factor.

Folic acid supplementation is indicated in patients with active hemolysis. Hematinic should be monitored in case of reticulocytosis or a worsening in anemia. In addition, vitamin B12 and iron are useful for RBC production because hemolytic episodes boost RBC turnover [14].

Patients receiving corticosteroids could be considered for anti-acid treatment if additional risk factors are present, such as concomitant use of NSAIDs or aspirin, active gastrointestinal bleeding, peptic ulcer, age over 60. A daily dose of more than 7.5 mg prednisolone for more than 3 months has been described to increase fracture risk due to osteoporosis. It is important to check an adequate daily intake of vitamin D (800 IU) and calcium (700–1200 mg) through diet or supplements if needed. Bisphosphonates in particular prevent fractures in patients receiving corticosteroids [98,99,100,101,102]. Infections may underlie secondary AIHA or be a complication of the treatment and worsen the course of AIHA. Therefore, it is necessary to test patients for infections and adapt measures for prevention, especially Mycoplasma pneumoniae, hepatitis B and C, HIV, EBV, CMV, parvovirus B19, and tuberculosis. Rituximab, a B-cell-depleting treatment, is associated to the reactivation of hepatitis B, therefore antiviral prophylaxis is recommended [103]. In case of splenectomy, vaccinations against Haemophilus influenzae, Meningococcus, and Pneumoccoccus species are recommended [104]. Risk of Pneumocystis jirovecii pneumonia is increased in patients receiving more than 20 mg corticosteroid/day for over one month. Trimethoprim-sulfamethoxazole, dapsone, atovaqone, or aerosolized pentamidine are licensed prophylaxis [105,106].

Finally, other supportive measures for CAD are avoidance of cold temperatures and symptomatic treatment of acrocyanosis including gloves and hand warmers [81].

## 4. Conclusions

AIHA is the most common autoimmune complication in CLL and its treatment is often correlated to disease status. Although corticosteroids are the standardized first-line treatment, in most cases it is necessary to start a new line of treatment. In this review, we analyzed the main chemo-immunotherapeutic agents used in this setting, firstly, rituximab. The recent development of targeted agents against BCR and BCL-2 generated excitement because they can be given orally, have few side effects, and are very effective both in progressive CLL and in situations such as AIHA. Nowadays, these targeted drugs are used after or together with steroids to manage AIHA. The challenge for the future could be to find the best drug combinations and identify the most appropriate therapeutic settings to optimize clinical benefit from these new agents.

## Figures and Tables

**Table 1:** Major published experiences on AIHA in CLL.

Study Type	N of CLL Patients	No. of AIHA	AIHA Type (Cold vs. Warm)	Main Findings	References
Single center, retrospective	1203	52	Warm 87% Cold 13%	Lymphocyte count more than 60 × 109/L, age > 65 years, and male gender are associated to increased rate of AIHA at CLL diagnosis IgG AIHA and concomitant diagnosis of AIHA and CLL are associated to better survival	Mauro, FR et al. 2000 [31]
Multicentric, prospective + retrospective	3150	129	Warm 89% Cold 11%	Age > 69 years, stage C, and R/R CLL are associated to AIC:	Barcellini, W. et al. 2006 [32]
Single center, retrospective	964	55	nk	Activated lymphocyte morphology, high levels of IgG and beta-2-microglobulin, and increased expression of CD38 and/or FMC7 are related with autoimmune complications	Duek, A. et al. 2006 [33]
Single center, retrospective	1737	41	nk	55% Treatment naïve CLL develop autoimmune diseases	Zent, C.S. et al. 2009 [34]
Single center, retrospective	960	49	nk	High lymphocyte count, short LDT, high serum B2M level, high ZAP-70 and CD38 expression are associated with AIC	Moreno et al. 2010 [35]
Multicentric, retrospective	1477	80	nk	Concomitant diagnosis of AIHA andCLL diagnosis is associated with shorter survival. The presence of positive antiglobulin test even without hemolysis was associated with worse outcome. Laboratory or clinical evidence of AIHA had a significant negative impact on the survival of patients with CLL.	Shvidel, L, et al. 2013 [36]
Single-centre prospective study	192	8 (4.2%)	nk	Older age and advanced stage of CLL are associated with AICs are.	Demir C. et al 2017 [37]
Multicentric, retrospective study	235	6 (2.6%)	Neg: *n* = 2 Warm: *n* = 2 Coombs positive, not known type = 2	Aggressive disease, poor prognostic features,, previous autoimmune phenomena or fludarabine front-line therapy are related with AIHA. BR is safe in previous AIHA orpositive DAT CLL patients.	Laurenti L. et al 2015 [25]
Single-centre retrospective study	795	27 (3.4%)	nk	Female gender, advanced stage disease, previous treatment, 11q deletion by FISH, CD38 positive and CAD are associated with autoimmune disease	Visentin A. et al 2017 [8]
Observational retrospective study	101	7 (6.9%)	nk	Patients with autoimmune cytopenia have less CLL BM infiltration and lesser response to treatment	Atef B. et al 2019 [38]
Critical review				The commonest AIC correlating with advanced disease and high biologic risk (del 11q, del17p, unmutated IGHV)	Visco C. et al. 2014 [39]

**Table 2:** Options for treatment of wAIHA.

Warm Autoimmune Hemolytic Anemia Waiha	References
First line	
Steroids (prednisone 1 mg/kg/day for 3–4 weeks; alternative dexamethasone 40 mg/day for 4 days, 2–6 cycles every 2–4 weeks)	[40,46,47,48]
Second line	
Rituximab 375 mg/m^2^ weekly × 4 weeks	[49,50]
New targeted drugs	
BTK inhibitors: ibrutinib and acalabrutinib	[51,52,53,54,55]
PI3K inhibitors: idelalisib	[56]
anti BCL2: venetoclax	[57,58]
Chemoimmunotherapic regimens:	
rituximab, cyclophosphamide and dexamethasone (RCD),	[59,60]
rituximab, cyclophosphamide, vincristine and prednisolone (R-CVP)	[61]
bendamustine and rituximab (BR)	[28]
Third or following line	
Alemtuzumab 30 mg × 3/week × 4–12 weeks	[51,62]
Cytotoxic immunesuppressors, such as cyclophosphamide, azathioprine, cyclosporine and mycophenolate	[63]
New-generation monoclonal antibodies: ofatumumab and obinutuzumab	[52]

**Table 3:** Options for treatment of cAIHA.

Cold Autoimmune Hemolytic Anemia Caiha	References
First line	
Rituximab 375 mg/m^2^ weekly × 4	[50,53]
Second line	
Rituximab plus Bendamustine 90 mg/m^2^	[54]
Rituximab plus Fludarabine 40 mg/m^2^	[55]
BTK inhibitors: ibrutinib and acalabrutinib	[64]
Upstream complement inhibition: eculizumab	[65,66]
